# Needs of Grandparents of Preschool-Aged Children with ASD in Sweden

**DOI:** 10.1007/s10803-019-03946-w

**Published:** 2019-03-01

**Authors:** Rano Zakirova Engstrand, Lise Roll-Pettersson, Mara Westling Allodi, Tatja Hirvikoski

**Affiliations:** 1 Department of Special Education, Stockholm University, 106 91, Stockholm, Sweden; 2 Department of Women’s and Children’s Health, Pediatric Neuropsychiatry Unit, Center for Neurodevelopmental Disorders at Karolinska Institutet (KIND), Karolinska Institutet, Stockholm, Sweden; 3 Habilitation & Health, Stockholm County Council, Stockholm, Sweden

**Keywords:** Grandchildren with autism, Grandparents’ needs, Sweden

## Abstract

Little is known about needs of grandparents of young children with autism in family and community settings. This study investigated perceived needs of grandparents of preschool-aged children diagnosed with ASD in the cultural context of Sweden. Participants were 120 grandparents of children enrolled into autism intervention programs provided by the public disability services in Stockholm. The Grandparents’ Needs Survey and the SDQ Impact supplement were used to collect data. Grandparents expressed most needs in topic areas of information and childcare. No significant relations were found between grandparents’ demographics and perceptions of needs; grandparents’ needs were predicted by their perceived burden. The findings provide insight into understanding of grandparents’ needs essential for planning and provision of quality family-centered early intervention services.

**Electronic supplementary material:**

The online version of this article (10.1007/s10803-019-03946-w) contains supplementary material, which is available to authorized users.

## Introduction

Autism spectrum disorder (ASD) is a neurodevelopmental disorder entailing difficulties with social interaction, communication and stereotypical behaviors and restricted interests (American Psychiatric Association 2013). Moreover, ASD is often associated with behavioral problems, eating and sleep disorders, depression and anxiety (Gillberg 2010). Therefore, the impact of ASD on family well-being is multidimensional (Derguy et al. 2015) and can adversely affect family quality of life (FQOL; Gardiner and Iarocci 2012; Tint and Weiss 2016). To alleviate demands that parents and siblings of a child with ASD may experience, grandparents’ role in supporting their grandchild and own adult offspring can be pivotal (Hastings 1997, 2008). Knowledge about grandparents’ needs, experiences, and the extent to which they are involved into everyday lives of families where a young child with autism is present can provide a more nuanced understanding of the impact of autism on a broader family system. This study will report findings on perceived needs of traditional (i.e. non-custodial) grandparents of young children with ASD in the cultural context of Sweden. To situate our findings, in the background section of the paper we will briefly review existing research on grandparents’ needs and experiences of having a grandchild with autism in a family system; outline factors that can influence grandparents’ experiences and needs, and, lastly, provide a short description of support services available to children with ASD and their families in Sweden.

### Grandparents’ Experiences and Needs when Having a Grandchild with ASD

Internationally available research on grandparents of children with ASD is limited (Hastings 2008; Hillman et al. 2016). Existing studies that examined views and perspectives of traditional grandparents of children with ASD have been mainly conducted in English-speaking countries. For instance, in the UK, Margetts et al. (2006) in a qualitative research project interviewed grandparents of children with ASD (aged 3–5 years) and found that grandparents described themselves as being parents to both to their own adult child and a grandchild, with some admitting feeling the double burden of taking care of two generations. Grandparents’ experiences were also described as “striving for answers”: they often struggled with the questions on how much they could help their adult children, or looking for answers on a possible cause of the disorder. In the USA, a quantitative study with 1870 non-custodial grandparents (Hillman et al. 2016) revealed that grandparents made substantial accommodations to support their grandchildren with ASD: they babysit, provided transportation and were engaged in teaching their grandchildren skills using various strategies. Grandparents also provided financial support to cover expenses for their grandchild’s therapies or other special needs (e.g. summer camps, legal support). In the Republic of Ireland, Prendeville and Kinsella (2018) qualitatively investigated grandparents’ role in supporting families of children with ASD aged 5–18 years. The researchers found that grandparents played an active role in strengthening their family system, and when necessary, they provided respite care to their grandchild with autism. In general, grandparents can be considered as an invaluable asset when providing family intergenerational support, and they can effectively ameliorate the impact of stressors affecting family quality of life (Kahana et al. 2015).

Findings reported in both quantitative and qualitative studies suggest that grandparents’ experiences of their relationships with their grandchild with ASD may be affected by a number of different factors: (a) grandparents’ characteristics, e.g., age, gender, lineage, level of education, employment status, health condition, and geographic proximity to the grandchild; (b) grandchild’s characteristics, e.g., age, severity of ASD symptoms; (c) grandparents’ relationships with the grandchild’s parents, and patterns of family functioning (D’Astous et al. 2013; Dougherty 2009; Glasberg and Harris 1997; Hillman et al. 2016; Margetts et al. 2006; Prendeville and Kinsella 2018; Sullivan et al. 2012). For instance, quantitative studies (Glasberg and Harris 1997; Hillman et al. 2016) and studies using qualitative research designs (D’Astous et al. 2013; Prendeville and Kinsella 2018) conducted in different cultural contexts showed similar results: in comparison with paternal grandparents, maternal grandparents had more knowledge about their grandchild; made more personal sacrifices to support their grandchild with ASD, shared the parents’ perspective of the child’s abilities, and provided more frequent emotional and instrumental care. In addition, Hillman et al.’s (2016) study revealed gender differences in provision of support to their families with grandmothers making more personal sacrifices than grandfathers, while grandfathers tended to provide more financial assistance for their grandchild’s needs compared to grandmothers. D’Astous et al.’s (2013) study reported that grandparents’ age and geographic proximity influenced their involvement with their grandchild: grandparents who were older and who lived at the greatest distance reported less contact with their grandchild with ASD. Studies have also pointed to the significance of intergenerational relationships in supporting the needs of the child with ASD. For instance, Glasberg and Harris (1997) suggested that discrepancies between grandparents’ and parents’ perceptions of the child with autism revealed in their study could be explained by grandparents’ unfamiliarity with the child. In the same vein, D’Astous et al. (2013) showed that intergenerational tension including criticism, poor communication, and limited understanding of their grandchild’s condition contributed to grandparents’ lesser level of support and involvement with their grandchild with ASD; whereas grandparents with good knowledge about autism, positive relationships with their adult children and solidarity with them on grandchild’s needs and supports were key factors affecting greater commitment to their grandchild with ASD.

Previous research has indicated that grandparents can have their own needs concerning their grandchild’s autism: they may have powerful emotional reactions to the diagnosis of ASD such as shock, anger, and grief (Kahana et al. 2015), and, therefore, may seek social support to deal with stressors in the family (Hillman 2007). Engaging into one-to-one interactions with their grandchild with ASD may require using physical strength for grandparents, which present more problems for them, especially for those who are in poor health (D’Astous et al. 2013). Grandparents may also seek information on their grandchild’s condition (Hillman 2007). For instance, in two qualitative studies (Hillman et al. 2017; Prendeville and Kinsella 2018) grandparents expressed most needs in obtaining information about ASD, and about strategies on how to manage grandchild’s tantrums and inappropriate behaviors, especially in public places. Other reported needs included a need for acknowledgement of grandparents’ role in supporting families by professionals (Prendeville and Kinsella 2018), and needs related to high costs for ASD treatments, grandchild’s selective eating, and lack of support from pediatricians (Hillman et al. 2017). Quantitative studies demonstrated that grandparents’ extensive information needs were associated with chaotic family functioning (Sullivan et al. 2012); other needs concerned grandparents’ seeking professional assistance to cope with their grandchild’s ASD (Hillman et al. 2016).

Despite these findings, research concerning the needs of grandparents of preschool-aged children with ASD is still scarce. Existing studies that investigated experiences and needs of grandparents included participants whose grandchildren with ASD ranged widely in age: from 1 to 19 years; however, these studies did not clearly report on differences in grandparents’ perceptions of needs based on their grandchild’s age. Previous research on needs of families with young children with disabilities showed that both parents and grandparents may face similar needs for information and support after they receive the news about their child’s diagnosis (Bailey and Simeonsson 1988; Vadasy et al. 1986). However, in comparison with the child’s parents, grandparents may not have the same resources or opportunities to obtain first-hand information about grandchild’s disability and/or available medical and educational services; moreover, grandparents’ informal social support system may not always overlap with those of parents (Vadasy et al. 1986). Coupled with grandparents’ initial emotional reactions to their grandchild’s condition, e.g. shock, grief and sadness, these factors could contribute to long-term negative effects of grandchild’s condition on grandparents’ health, which may diminish the effectiveness of supports provided to parents (Hastings 1997). Increased knowledge pertaining to grandchild’s disability can enhance grandparents’ ability to provide emotional and instrumental support to the whole family as well as help to cope with challenges that childhood disability may bring to family’s everyday functioning (Seligman and Darling 1997). Furthermore, a deeper understanding of the needs expressed by grandparents is necessary for practitioners when planning and providing high quality family-centered early intervention services.

To the best of our knowledge, there is no study available that explicitly describes unique needs of grandparents of preschool-aged children with ASD in the context of Nordic countries. In Sweden, research on grandparents of children with disability, in general, and of children with ASD, in particular, is virtually non-existent. Swedish empirical research on older generation in the area of social sciences is mostly limited to investigation of living conditions of older people (65 +) or effects of psychosocial factors on their well-being in the context of the Swedish welfare system (e.g. Bask 2015; Heap 2016). Other existing studies involving older generation have taken a comparative perspective in investigation of various issues related to grandparenting in several European countries, including Sweden (Di Gessa et al. 2016a, b; Muller and Litwin 2011). This line of research suggests that those grandparents who look after their grandchildren experience better health than those who do not (Di Gessa et al. 2016a). This research has also shown that grandparental childcare has differential effects on men’s and women’s health outcomes (Di Gessa et al. 2016b), with women experiencing lower levels of psychological well-being (Muller and Litwin 2011). Literature on intergenerational relations has indicated that a typical Swedish household includes two generations, whereas three-generation households are very rare (Fors and Lennartsson 2008). Several authors argue that the strength of intergenerational relations among family members in contemporary Swedish families is rather weak due to (a) individualistic values held by the majority of Swedish families, and (b) the strong welfare state system that supports both children and the elderly (Bask 2015; Kolk 2014). However, geographic proximity of older generation to their adult children and grandchildren has been noted as the main factor influencing contact and provision of care in Swedish families (Kolk 2017). Although instructive, these studies did not consider including having a grandchild with disability as an additional variable into analyses; included grandchild’s characteristics were mostly limited to age and number but not disability status. The present study, therefore, intends to contribute to the field by addressing this gap in the literature and aims to explore grandparents’ perceived needs in relation to having a young grandchild diagnosed with ASD in the context of the Swedish support system.

### Overview of Supports and Services for Children with ASD in Sweden

In Sweden, the social welfare system ensures free access to universal child health care with routine screening for developmental delays (Idring et al. 2012) and comprehensive assessment for ASD (Rai et al. 2012). Regular routine check-ups and vaccinations are conducted at Child Health Centers (CHCs) covering almost 99% of all children in Sweden (Wettergren et al. 2016). If a young child is suspected for ASD during routine screening, the child is referred to specialized Child and Adolescent Psychiatry Clinics (CAPCs) for further diagnostic assessment. Children diagnosed with ASD and their families are entitled to support services provided jointly by the state, municipalities, and regional county councils under several national legislative acts: (1) Health- and Medical Services Act (2017:30); (2) Act Concerning Support and Service for Persons with Certain Functional Impairments (1993:387, known as *LSS*, in Swedish); (3) Social Insurance Code (2010:110); (4) Social Services Act (2001:453); (5) Education Act (2010:800); (6) Special Transport Services Act (1997:736), and (7) Act on Housing Adaptation Grants (1992:1574). While provision of healthcare services falls under jurisdiction of regional county councils, provision of educational, social or transportation services falls under jurisdiction of municipalities. All services are free of charge for children with ASD regardless of their socio-economic status, ethnic/cultural background, immigration status, place of residence (urban or rural), or service provider (public or private) (Swedish National Board of Health and Welfare [SNBHW] 2014).

Publicly funded healthcare services for young children with autism are provided through Child disability services centers (known as child habilitation centers), and include behavioral interventions, speech therapy, physiotherapy, and occupational therapy (Spjut Jansson et al. 2016); psychoeducation and support to child’s family members (parents, siblings; in some cases, to extended family members such as grandparents), as well as to preschool staff (Barnevik Olsson 2016). Evidence-based interventions grounded in the principles of applied behavior analysis—including both focused (non-intensive) and comprehensive (e.g. Early Intensive Behavioral Intervention [EIBI])—are carried out in community based preschool settings by preschool staff under supervision of professionals from habilitation centers (Roll-Pettersson et al. 2016). There are only a few private healthcare providers of services for children with ASD in Sweden delivering evidence-based treatments such as EIBI. All families regardless of their socio-economic or cultural background can apply for participation in an EIBI program; however, preschools’ refusal to participate in the program as well as long-waiting lists for up to 8–12 months were mentioned as main barriers hindering children and their families from obtaining timely support (Wenneborg, Personal Communication, 21 January 2019). Regardless of the type of the service provider other barriers to provision of quality services and evidence-based treatments have been identified: low levels of ASD knowledge among preschool teachers working in mainstream classroom settings (Zakirova Engstrand and Roll-Pettersson 2014); lack of knowledge and allegiance towards EIBI (Långh et al. 2017), and lack of collaboration between various agencies responsible for provision of services for children with ASD and their families in general (SNBHW 2018; Westman Andersson et al. 2017).

### Research Questions and Hypotheses

The present study draws on the findings on family needs from existing literature and uses the following definition of family needs provided by Simeonsson (1996): “The needs that families express can be seen as broader expectations reflecting what families expect in the form of supportive services” (p. 205).The study sought to answer the following research questions and hypotheses:

1. What are the needs of grandparents of a young grandchild with ASD in relation to information, family and social support, financial support, explaining to others, child care, professional support, and community service? It was hypothesized that grandparents would identify their needs at least in one of the topic areas as mentioned above.
2. What are the associations between grandparents’ socio-demographic characteristics and their needs when having a grandchild with ASD? It was hypothesized that at least one of the grandparents’ characteristics (age, gender, relation to grandchild with ASD, level of education, employment status, geographic proximity to grandchild with ASD, frequency of meeting grandchild, or health condition) would predict grandparents’ perceived needs.
3. What are the associations between grandparents’ perceptions of needs and their perceptions of (a) grandchildren’s difficulties and (b) impact these difficulties might have on grandchildren’s everyday life? It was hypothesized that grandparents’ perceptions of grandchild’s difficulties and impact of these difficulties on grandchild would predict grandparents’ needs.

## Method

### Design and Setting

This study is part of a larger research project carried out in collaboration between Department of Special Education, Stockholm University, and the Autism Center for Small Children (ACSC) at Habilitation & Health, Stockholm (Stockholm County Council publicly funded disability services). The study was approved by the Regional Ethics Board in Stockholm (2017/286-31/5). As part of the comprehensive psychoeducational program for families of young children with ASD (0–6 years old), the ACSC offers a 1-day course for grandparents. The objectives of the course are: (1) to increase grandparents’ knowledge about ASD and difficulties associated with the disorder; (2) to provide information on how grandparents can help to support children’s positive development; (3) to discuss grandparents’ role in supporting their own adult children, and (4) to provide forum for exchanging experiences with other course participants. The course is offered to 2 or 3 groups of grandparents per semester. Group sizes usually comprise of 30–35 maternal and paternal grandparents, including step-grandparents.

### Recruitment and Participants

Data were collected from grandparents whose grandchildren with ASD were enrolled into the ACSC’s intervention programs at four separate full-day psychoeducational workshops held in March, May, October, and November 2017. Grandparents were asked to come to the workshop venue 40 min before the course started in order to obtain information about the aims of the study and to answer survey questions if grandparents agreed to participate. A brief information about the study was also provided in the course catalogue. As an incentive for participating in the study, the grandparents were served free breakfast. During the breaks, all grandparents were invited to tea, coffee and fruit regardless of their participation. Upon arrival, the grandparents were first verbally informed about the aims of the study, and then each of them received a package containing: (1) a letter describing the aims of the project, the procedure, and information about participant’s right to withdraw at any time, and anonymity and confidentiality of data; (2) a consent form; (3) questionnaires in A4-sized paper. Overall, 135 traditional (non-custodial) grandparents participated in the course; of them 120 (n = 120; 89%) agreed to participate in the study. Written consents were obtained from these grandparents; all of them were included into analyses.

### Measures

Measures used in this study include a demographic survey, the Grandparents’ Needs Survey, and the Swedish version of the impact supplement to the Strength and Difficulties Questionnaire (SDQ-Swe; Malmberg et al. 2003) based on Goodman’s (1999) original extended version of the SDQ.

#### Demographic Survey

The demographic survey is a modified and translated version of the demographic section of the Needs Survey for grandparents of children with disabilities developed by Dougherty (2009). It consists of 11 questions and asks participants to indicate their gender, age, mother tongue (as proxy for ethnicity), level of education, employment, perceived health, urban or rural setting of grandparent residence, and geographic proximity of grandparent residence to grandchild with ASD. The survey also asks questions on grandchild’s age and sex.

#### Grandparent Needs Survey

Grandparent Needs Survey is a modified version of the Needs Survey for Grandparents of Children with Disabilities (Dougherty 2009), which was based on the Family Needs Survey (FNS; Bailey and Simeonsson 1988) and its revised version (Bailey and Simeonsson 1990). The instrument is a 40-item scale (0—“no”; 1—“maybe”; 2—“yes”) with items grouped into seven topic areas: (1) information; (2) family and social support; (3) financial support; (4) explaining to others; (5) child care; (6); support from professionals; (7) community services. The Swedish translation of the FNS was used earlier in the studies that investigated the needs of parents of children with various types of disabilities (Roll-Pettersson 1992, 2003; Granlund and Roll-Pettersson 2001). For the present study several adjustments were made in the available Swedish translation, where some words and phrases were either omitted or changed in accordance with the currently used terminology in Sweden. However, back translation was not done. For the purposes of the study, we retained most of the items comprising the Needs Survey for Grandparents of Children with Disabilities (Dougherty 2009). However, we excluded several items as either not applicable to the Swedish context, or as overlapping with other items in the survey. (A detailed description of the used items are available upon request from the first author). We also excluded open-ended questions as part of the original instrument due to a planned interview study within the framework of the project. In the present study, the internal consistency of the Grandparent Needs Survey (of the scale) and its seven subscales was calculated; for the subscales Cronbach’s α were: information 0.80; family and social support 0.88; financial support 0.90; explaining to others 0.87; child care 0.74; support from professionals 0.79, and community services 0.84. Cronbach’s alpha coefficient for the whole scale was 0.93.

#### Strengths and Difficulties Questionnaire—Swedish Version

To collect additional descriptive data on children characteristics as perceived by the grandparents in terms of perceived difficulty, chronicity, distress and social impairment, as well as to measure grandparents’ perceived burden of grandchildren’s difficulties for them or for the entire family, we used the Swedish translation of the parent-specific impact supplement included into the extended version of the Strength and Difficulties Questionnaire (SDQ; Goodman 1999). The original version of the SDQ is the 25-item instrument designed for a brief screening for behavioral attributes of the child (Goodman 1997); it has three versions: self-report (by children and teenagers aged between 11 and 16 years), parent, and teacher. The parent-specific version of the impact supplement scale asks parents to rate if their child has difficulties in such areas as emotions, concentration, behavior, or an ability to get on with other people; if yes, it then asks to rate the chronicity of these difficulties, overall distress and impairments in four domains of child’s social life: home life, friendship, classroom/learning environment, and leisure activities. In addition, it asks to rate parents’ perceived burden on them or their family. Responses from the impact supplement scale may illicit important information predictive of future help-seeking behavior and service use (Goodman 1999). For the purposes of the study, slight modifications to the instrument’s wording were made, e.g. the phrase *your child* had been changed into *your grandchild; classroom environments* were changed to *learning environments*. We followed the scoring guidelines provided by Goodman (1999). The responses to the first item—perceived difficulties—were rated on a four-point scale: 0 = “no”; 1 = “minor”; 2 = “definite”; 3 = “severe”. Chronicity was rated on a four-point scale: 1 = “less than a month”; 2 = “1–5 months”; 3 = “6–12 months”; 4 = “over a year”. The impact score was calculated as a sum of the responses to five items: distress and social impairment when each impact item was rated on a three-point scale: 0 = “not at all/only a little”; 1 = “quite a lot”; 2 = “a great deal”. The total impact score measures the extent to which children’s’ difficulties are perceived as distressful and interfering children’s everyday life. Burden was rated on a four-point scale: 0 = “not at all”; 1 = “only a little”; 2 = “quite a lot”; 3 = “a great deal”. Previous validity studies of the Swedish version of the extended SDQ based on parents’ ratings reported good psychometric properties for the Impact score with Chronbach’s α for community sample 0.87, and for clinical sample 0.76 (Malmberg et al. 2003). In the present study, the internal consistency (Chronbach’s α) for the Impact score scale was 0.85. The mean scores for each item comprising the Impact supplement scale were calculated (see Table A, Supplementary Online Material).

### Data Analysis

Demographic data were analyzed descriptively using the SPSS software program, version 24. Descriptive statistics were also used to analyze the participants’ responses to the Grandparents Needs Survey. To test the second and the third hypotheses, we created two multiple regression models. In the first model we included eight demographic variables (i.e. age, gender, lineage, level of education, employment status, health condition, geographic proximity to grandchild with ASD, frequency of meeting grandchild) as independent variables. In the second model, the included independent variables were items comprising the SDQ Impact supplement scale (perceived difficulties, total impact score, and perceived burden on grandparent or family). In both models, the dependent variable was grandparents’ needs (sum score of all items). Missing data were treated by using the pairwise deletion approach commonly used when performing correlation analyses (Graham 2009). This approach was also chosen as it allows using all available data by discarding cases on an analysis-by-analysis basis (Peugh and Enders 2004; Howell 2007).

## Results

### Demographic Characteristics

Demographic characteristics of the participants are presented in Table 1. While all grandparents were traditional (non-custodial) grandparents, six grandparents (5%) described themselves as step-grandparents, none reported being foster grandparents, and two participants (1.7%) did not specify their relationship to the grandchild. The majority of the participants were Swedish-speaking (90.1%) and more than a half (63.3%) were women. More than a half of the participants (62.5%) reported living in a large city. Almost 85% of the respondents reported their health condition as “good”. Of 119 grandparents (missing data *n* = 1), 9 (7.6%) reported having two grandchildren diagnosed with ASD.

**Table 1:** Demographic characteristics of grandparents

Grandparent characteristics (n = 120)	n (%)
Sex	
Female	75 (63.6)
Male	43 (36.4)
Missing data n = 2	
Age	
40–55	5 (4.2)
56–65	45 (38.1)
66–75	63 (53.4)
Older than 75	5 (4.2)
Missing data n = 2	
Relation to grandchild	
Maternal grandmother	36 (30.5)
Maternal grandfather	21 (17.8)
Step maternal grandmother	1 (0.8)
Step maternal grandfather	3 (2.5)
Paternal grandmother	35 (29.7)
Paternal grandfather	18 (15.3)
Step paternal grandmother	2 (1.7)
Step paternal grandfather	–
Other	2 (1.7)
Missing data n = 2	
Mother language	
Swedish	100 (90.1)
Non-European	5 (4.5)
European	6 (5.4)
Missing data n = 9	
Education	
High school	11 (9.4)
Upper high school	41 (35.0)
University or university college	65 (55.6)
Data missing n = 3	
Occupation	
Full-time job	18 (15.1)
Part-time job	12 (10.1)
Hourly work	1 (0.8)
Has own company	2 (1.7)
Unemployed	2 (2.7)
Retired	72 (60.5)
Retired, employed part-time	12 (10.1)
Missing data n = 1	
Perceived health condition	
Good	94 (84.7)
Moderate	16 (14.4)
Poor	1 (0.9)
Missing data n = 9	
Geographic proximity to grandchild (km)	
< 10	7 (6.3)
10–25	60 (53.6)
26–50	23 (20.5)
51–100	3 (2.7)
101–500	11 (9.8)
> 500	7 (6.3)
Lives together with grandchild and other family members	1 (0.9)
Missing data n = 8	
Place of residence	
Countryside/small town (< 10 000 residents)	23 (20.5)
Medium-size town (10 000–60 000 residents)	19 (17.0)
City (> 60 000 residents)	70 (62.5)
Missing data n = 8	
Frequency of contact grandchild	
Every day	3 (2.7)
Every week	65 (58.6)
Every month	28 (25.2)
Less often than monthly	15 (13.5)
Missing data n = 9	

Each characteristic is provided with a valid number of responses

Grandparents reported the demographic details for their 124 grandchildren (data missing *n* = 5), of whom 30 (24.2%) were girls and 94 (75.8%) were boys. The mean age was 4.07 years, (*SD* = 0.98; *n* = 128, missing data *n* = 1). Table 2 presents grandchildren’s characteristics based on the SDQ Impact supplement scale as reported by their grandparents.

**Table 2:** Characteristics of grandchildren with ASD as reported by their grandparents (n = 120) on the Impact Supplement scale items of the SDQ-Swe

Grandchildren characteristics	n (%)
Perceived difficulties	
No difficulties	1 (1)
Yes, minor difficulties	45 (44.1)
Yes, definite difficulties	49 (48.0)
Yes, severe difficulties	7 (6.9)
Missing data n = 18	
Chronicity	
1–5 months	1 (1.0)
6–12 months	7 (6.9)
More than a year	94 (92.2)
Missing data n = 18	
Impact score	
1. Distress	
Not at all/only a little	67 (71.3)
Quite a lot	25 (26.6)
A great deal	2 (2.1)
Missing data n = 26	
2. Home life	
Not at all/only a little	54 (55.7)
Quite a lot	37 (38.1)
A great deal	6 (6.2)
Missing data n = 23	
3. Friendships	
Not at all/only a little	40 (44.4)
Quite a lot	34 (37.8)
A great deal	16 (17.8)
Missing data n = 39	
4. Learning environments	
Not at all/only a little	43 (48.9)
Quite a lot	33 (37.5)
A great deal	12 (13.6)
Missing data n = 32	
5. Leisure activities	
Not at all/only a little	52 (59.1)
Quite a lot	24 (27.3)
A great deal	12 (13.6)
Missing data n = 32	
Perceived burden on grandparent or family	
Not at all	51 (50.5)
Only a little	41 (40.6)
Quite a lot	7 (6.9)
A great deal	2 (2.0)
Missing data n = 19	

Each child characteristic is provided with a valid number of responses

### The Needs of Grandparents of a Young Grandchild with ASD

**Fig. 1:**
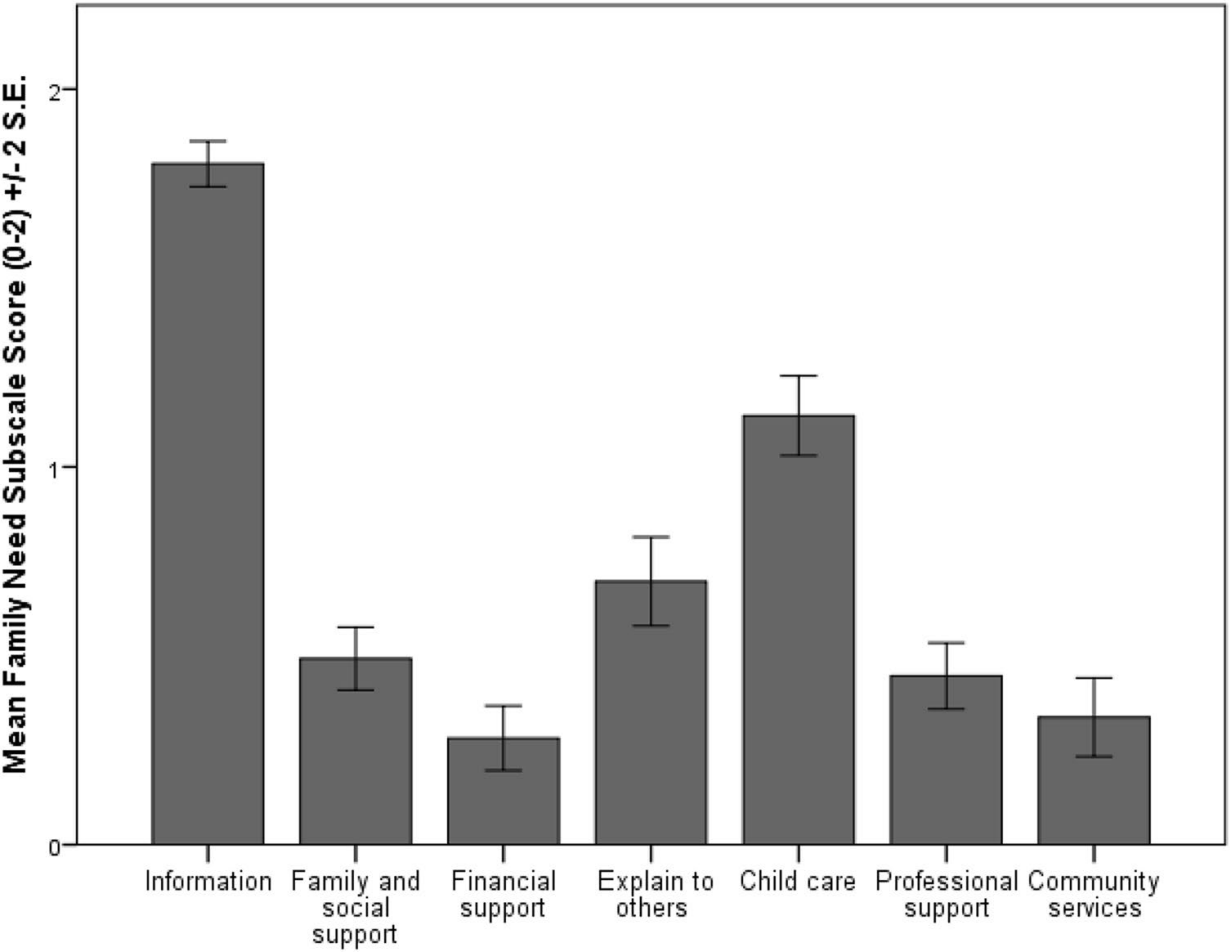
Mean scores of the Grandparents Needs Survey’s subscales

Figure 1 shows the results of descriptive analyses based on the seven categories in the Grandparents Needs Survey. Grandparents expressed most needs for information (*M* = 1.80, *SD* = 0.33) followed by needs in topics related to childcare (*M* = 1.14, *SD* = 0.58), explaining to others (*M* = 0.70, *SD* = 0.64), needs for family and social support (*M* = 0.49, *SD* = 0.45), professionals support (*M* = 0.45, *SD* = 0.47), community services (*M* = 0.34, *SD* = 0.56). The least expressed needs were for financial support (*M* = 0.28, *SD* = 0.47).

The mean scores for each survey item are shown in Table B (Supplementary Online Material). In the category *information*, the results show that the grandparents expressed most needs for learning more about strategies to help their grandchildren develop skills, followed by needs to obtain more information about how to handle grandchild’s behavior, and information about grandchild’s ASD. In the category *family and social support* analyses showed that grandparents needed most support in three areas: talking to grandchild’s parents about concerns related to the grandchild with ASD, helping the family to discuss problems and reach solutions, and helping to support each other in the family during difficult times. Within the *financial support* category grandparents reported most needs in one area: getting any special equipment for grandchild’s needs. Grandparents’ needs within the category *explaining to other* showed most expressed needs in finding reading material about families who have a grandchild like theirs. This was followed by a need to get assistance in explaining the grandchild’s ASD to other children, and knowing how to respond when friends, neighbors or strangers ask about the grandchild with ASD. In the category *childcare* the grandparents reported most needs about learning how to provide adaptive play or recreation experiences for their grandchild. The least expressed need was locating an appropriate childcare facility for their grandchild with ASD (e.g. preschool). The results for the category *professional support* show that grandparents expressed most needs in two areas: learning how to communicate with teachers and other professionals regarding their grandchild with ASD, and accessing family counselling for parents and grandparents. The least expressed need was getting help in meeting with a leader of religious faith (e.g. priest, imam or rabbi). The results for the *community service* category shows that grandparents needed help in finding a family doctor or a specialized medical doctor for their grandchild with ASD who could understand their grandchild’s needs (please see Table B, Supplementary Online Material).

### Relationships Between Grandparents’ Background Variables and Perceived Needs

The results of multiple regression analyses revealed no significant associations between grandparents’ demographic variables and their perceptions of needs (Table 3).

**Table 3:** Correlations matrix of variables and regression results: predicting grandparents’ perceived needs on grandparents’ socio-demographic characteristics

Variable	1	2	3	4	5	6	7	8	9	B	SE B	β	Sig.	95% CI	
														Lower	Upper
1. Total Needs score	–									43.27	9.90		0.000		
2. Age	− 0.087	–								− 1.58	2.64	− 0.08	0.552	− 6.81	3.66
3. Gender	− 0.075	0.145	–							− 1.26	2.77	− 0.05	0.649	− 6.76	4.23
4. Relation to grandchild	− 0.113	0.188	0.194	–						− 0.60	0.62	− 0.10	0.346	− 1.83	0.65
5. Education level	− 0.103	0.073	0.059	− 0.054	–					− 1.50	2.03	− 0.08	0.463	− 5.51	2.53
6. Employment status	− 0.033	0.641	0.101	0.147	0.046	–				0.18	0.80	0.03	0.826	− 1.40	1.74
7. Health condition	0.100	0.150	0.050	0.020	0.278	0.096	–			3.15	3.82	0.09	0.412	− 4.42	10.72
8. Proximity	− 0.115	0.057	0.046	0.116	0.098	− 0.037	− 0.021	–		− 0.60	1.27	− 0.06	0.647	− 3.10	1.93
9. Frequency of contacts	− 0.150	0.071	0.151	0.098	0.013	− 0.009	− 0.062	0.642	–	− 1.40	2.29	− 0.08	0.543	− 5.94	3.15

*R*^2^ = 0.06, *F* = 0.74, *CI* confidence interval

### Relationships Between Grandparents’ Needs and Perceptions of Grandchild’s Difficulties

No associations were found between grandparents’ perceptions of needs and (a) their perceptions of grandchild’s difficulties, or (b) perceived impact that these difficulties might have on grandchild’s everyday life (Table 4). The regression analyses revealed that grandparents’ perceptions of needs were predicted by their perceived burden (β = 0.352, p < .05) (Table 4). Given the importance of the gender variable from previous research, an additional analysis using a non-parametric statistical technique—the Kruskal–Wallis test—was performed to explore the impact of the gender variable on perception of burden; results revealed no statistically significant difference in levels of perception of burden of women (*Md* = 1, n = 65) and men (*Md* = 1, n = 34): χ^2^ (1, n = 99) = 0.011, p = .918.

**Table 4:** Correlation matrix of variables and regression results: predicting grandparents’ perceived needs on SDQ Impact supplement items: perceived difficulties, impact score, and burden

Variable	1	2	3	4	B	SE B	β	Sig.	95% CI	
									Lower	Upper
1. Total needs score	–				24.23	6.60		0.000	11.06	37.40
2. Perceived difficulties	0.205*	–			1.89	2.86	0.09	0.510	− 3.79	7.58
3. Total impact score	0.210*	0.598**	–		− 0.50	0.90	− 0.10	0.567	− 2.21	1.22
4. Perceived burden	0.324**	0.495**	0.729**	–	6.90	3.16	0.35	0.033	0.56	13.17

*R*^2^ = 0.11, *F* = 3.05*, *CI* confidence interval. *p < .05 (2-tailed), **p < .001 (2-tailed)

## Discussion

To our knowledge, this is the first study that has explored grandparents’ perceived needs in relation to their young grandchild diagnosed with ASD in the context of the Swedish support system. Overall, results indicate that grandparents expressed most needs with in the subscale pertaining to information, followed by needs in topics related to childcare, thus, confirming our first hypothesis; the least expressed needs were for financial support. Before we discuss the results, we would like to point to some observations made on the demographic characteristics of the study sample. The participants consisted of 120 traditional maternal and paternal grandfathers as well as step-grandparents. One important observation is that in relation to grandchild with ASD, our sample did not differ much in a number of maternal and paternal grandmothers (n = 36 and n = 35, respectively), or in a number of maternal and paternal grandfathers (n = 21 and n = 18, respectively). Previous research with grandparents of children with ASD reported that their samples included more maternal grandparents than paternal grandparents (Glasberg and Harris 1997; Hillman et al. 2016, 2017; Margetts et al. 2006; Prendeville and Kinsella 2018), or had a fewer number of paternal grandmothers compared to maternal grandmothers (Sullivan et al. 2012). In addition, the majority of grandparents reported residing in large urban settings, which is not surprising: their grandchildren received support services at the Habilitation & Health, Stockholm County Council, located in the capital city of Sweden, Stockholm. Another observation is that our participants represent culturally and linguistically homogenous group: only 11 grandparents (9.9%) reported having their mother language other than Swedish (Table 1), which is somewhat lower than expected, given the fact that modern Sweden is a multicultural society with nearly 20% of its total population that consists of nationalities from almost 200 countries (Tavallali et al. 2014). The overwhelming majority of our sample (84.7%) perceived their health condition as good, thus, supporting previous findings on self-rated health among grandparents in Europe (DiGessa et al. 2016a). We did not inquire about grandparents’ income to ensure our participants’ integrity, but also because we thought that the items in the Financial support subscale of the Grandparents Needs survey would provide sufficient information in regard to participants’ financial situation.

The analyses of the Grandparent Needs survey demonstrate that grandparents’ strongest perceived needs were in the area of information, especially, concerning how to help grandchildren develop skills, how to handle problem behavior, and the effect of autism on grandchildren. This finding is similar to the results reported by Dougherty (2009) where a need for information was the greatest among grandparents of children with disabilities in the US context. Our findings are consistent with previous studies (Hillman et al. 2017; Prendeville and Kinsella 2018). For instance, in their qualitative study, Hillman et al. (2017) reported that two of the most important priorities for grandparents were needs for information about how to handle grandchild’s behavior, and for information about ASD. These needs were preceded by the need for more information about how to help the grandchild develop skills as well as learn to manage disruptive behavior and tantrums (Hillman et al. 2017). Notably, as in the present study, these findings reported by grandparents echo the results reported by parents of children with ASD in Derguy’s et al. (2015) study, for whom information about ASD and knowledge about management of child behavior were key areas of perceived needs.

However, in the information topic area, there were several notable differences between participants’ responses in our study and those reported by Dougherty (2009), mostly, on the level of individual items: while Dougherty (2009) reported grandparents’ need for information about laws and rights regulating provision of services as the greatest, the grandparents in the present study ranked this need as least important. Similarly, unlike Dougherty’s (2009) findings, the topic area of financial support was among the least prioritized needs for the grandparents in our study. Differences in the results could be attributed to several factors. Firstly, the present study included grandparents of preschool-aged children with a clinical diagnosis of ASD, while Dougherty’s (2009) study included grandparents of children with various ages (0–21), with disabilities not limited to ASD only, but also other types, e.g. Down syndrome, visual impairment, spina bifida, cerebral palsy. Secondly, this finding could be explained by differences in provision of support services in two distinct cultural contexts of Sweden and of the USA. Socio-cultural differences could as well explain several other findings in the present study in relation to findings from previous research on grandparents’ needs. For instance, in our study the least expressed need was found in the topic area of *professional support*, namely, for item *Getting help from a leader of religious faith*, when 99.1% of all participants (n = 115) reported no need of support in comparison with only one participant (0.9%) who disclosed a definite need of support in this area. (Of interest, this participant self-reported as not being of Swedish origin based on language as proxy for ethnicity). This result is consistent with evidence from previous research on religion, mental health, and well-being conducted with older adults in Sweden, indicating very low rates of religious involvement among Swedes with only 1% of those who sought support in religion to cope with stress (Cederblad et al. 1995; Koenig and Larson 2001; Cohen and Koenig 2003). The lack of need for religious support expressed by the overwhelming majority of grandparents in the present study somewhat supports Dougherty’s (2009) finding showing that 67.2% of grandparents residing in the US state of Kentucky reported no need in getting assistance to meet with a leader of their religious faith. Similarly, Hillman et al. (2016) noted that in their sample, only 24% of grandparents who coped poorly with their grandchild’s ASD sought professional assistance from clergy. Interestingly, these results, including our study, contrast with the finding reported in a study conducted in the USA 30 years ago (Vadasy et al. 1986) when the majority of grandparents of children with disabilities described their involvement into religious groups as active with 71% participating in church activities at least once a month, and with 67% reporting that their religion helped them understand and accept their grandchild with disability. Dissimilar results revealed by this earlier study by Vadasy et al. (1986) and by the most recent ones (e.g. Dougherty 2009; Hillman et al. 2016) including the present study, could be explained by possible cohort effects. It is possible that within these cultural contexts religiosity in general has become less strong over time, and that for grandparents of children with ASD other sources of support may have become more salient than seeking support from religious leaders. Indeed, as Hillman et al.’s (2016) study showed, of those grandparents who did not cope well with their grandchild with ASD had sought professional help by turning to support groups (24%), psychologists (17%), social workers or other therapists (17%). In the present study, a need for *Learning how to communicate with teachers and other professionals regarding grandchild with ASD* together with a need for *Accessing family counselling for parents and grandparents* were the most prioritized needs reported by grandparents in the category Professional Support (see Table B, Online Supplementary Material).

Another finding in the present study that could be attributed to cross-cultural differences concerns the item *Locating an appropriate childcare facility for their grandchild with ASD* as the least reported need in the Childcare category. Consistent with previous research on cultural variations on the prevalence of grandparental childcare across European countries (Di Gessa et al. 2016; Muller and Litwin 2011), this finding might suggests that, generally, in Sweden grandparents rely on formal childcare provision ensured by the state’s welfare policy, and therefore, they might not feel a need to look for other appropriate childcare facilities for their grandchildren. As Di Gessa at al. (2016) demonstrated, availability of formal public childcare provision coupled with women’s access to paid work is associated with provision of intensive grandparental childcare (i.e. at least 15 h/week). For instance, the authors showed that in Sweden, a country characterized with high level of formal public childcare provision, only 3.6% of parents received help from child’s grandparents compared to other European countries with lower levels of formal childcare provision, where parents had to rely more on their own parents in looking after children.

The present study also aimed at investigating possible relationships between grandparents’ socio-demographic characteristics and their perceived needs. The findings did not confirm our second hypothesis: there were no significant results in perceived needs based on grandparent’s relation to grandchild (i.e. lineage), gender, employment status, or educational background, or frequency of meeting grandchild (see Table 3). There were also no associations found between perceptions of needs and grandparents’ geographic proximity to the child as well as grandparents’ self-rated health. Based on previous research, we expected that at least one of the above-mentioned demographic variable would be associated with grandparents’ perceptions of needs. Indeed, studies with grandparents of children with various types of disabilities showed that both grandparents’ better health and higher education level correlated positively with lower level of needs (Dougherty 2009); whereas in Vadasy et al.’s (1986) study, the grandparents who had a university degree expressed high level of needs for information about availability of various support services for their grandchildren and families. Previous research has also shown the importance of including the gender variable into analyses when studying family needs, with results indicating that women tend to express more needs than men in relation to their child with disability. For instance, the Wang and Michaels’s (2009) study on parental needs revealed that mothers had greater needs compared to fathers on the information subscale. Yet, the results of the present study demonstrated no observed relationships between the gender variable and perception of needs among grandparents, adding to inconclusiveness of research evidence on the influence of demographic factors on family needs. We suggest that the results of the present study could be explained by the potential effects of the Swedish social welfare system characterized by gender equality (Kolk 2014) and high level of formal public childcare provision (Di Gessa 2016), indicating that equal access to social services, health care and childcare provision ensured by the state could be accounted for insignificant role of grandparents’ socioeconomic status, gender, lineage, or other demographic factors in providing grandchild care in Sweden. Future research could consider including broader societal factors such as social norms and existing policies regulating services and support to both young and elderly as moderating variables in models of analysis to understand their possible influence on grandparents’ perceptions of needs when having a grandchild with ASD in various cultural contexts.

Another explanation to the finding could be potential selection bias (Šimundić 2013) suggesting that grandparents who participated in our study might not be fully representative of grandparents of young children with ASD in Sweden. Indeed, as we have mentioned earlier, the participants in our study were not representative of grandparents with immigrant background. Besides, our sample represented those families whose children and their parents resided and obtained support services in the greater Stockholm area; moreover, these grandparents were invited to participate in the seminar as part of the service package specifically aimed to address grandparents’ informational needs related to ASD. Given that the overwhelming majority of the grandparents in our sample (84.7%) described their health as good, it is possible that needs of those grandparents who could not attend the seminars due to poor health would differ from those reported in the present study. Indeed, according to the data from the Swedish national public health survey (Public Health Agency of Sweden 2016), 62% of older people aged 65–84 years reported their health as “good” or “very good”. Furthermore, based on previous research (D’Astous et al. 2013), one could argue that it is possible that grandparents in our study chose to participate in the seminars because they had closer and more committed relationships with their adult children and grandchildren with ASD and, therefore, were more involved into grandchild care and wanted to gain more knowledge about how to support their families in the most optimal way. Further research involving more heterogeneous and representative samples selected by using random methods is required to examine possible relations between grandparents’ needs and socio-demographic characteristics in diverse subgroups of grandparents of young children with ASD, including those with ethnic and cultural background other than Swedish and those residing in geographic areas other than the greater Stockholm region. Qualitative research designs could shed light on how patterns of relationships between grandparents and their adult children might affect grandparents’ level of involvement with grandchild with autism, which in turn could help understand further the unique needs of these grandparents.

The results of our study revealed no observed associations between grandparents’ perceived needs and their perceptions of grandchildren’s difficulties or impact these difficulties might interfere with their grandchild’s social life (total impact score) as measured by the SDQ Impact supplement scale. The most plausible explanation to this finding could be grandparents’ difficulty to describe their grandchildren’s functioning in everyday life. In this study, all but one participating grandparents reported not living together with their grandchildren in the same household. It is not surprising, given the fact that in Sweden family support seldom involves multi-generational co-residence (Muller and Litwin 2011). Another possible explanation to this finding could be the instrument chosen for data collection. The SDQ Impact supplement scale was primarily designed to be completed by immediate family members, i.e. parents, and by teachers (Goodman 1999) who meet and interact with children on a daily basis, and who can make reliable observations of child’s behavior throughout the day, suggesting that the instrument might not be entirely suitable for use with non-custodial grandparents, as they do not always meet their grandchildren on a daily basis and, therefore, lacked information on grandchild’s social functioning. Future researchers may want to develop and use more sensitive measures tailored for this population of grandparents, as well as to use instruments designed specifically for children with ASD in order to assess their functioning abilities and disabilities in various contexts. For instance, Bölte et al. (2018) recently published the Brief ICF Core Sets for ASD based on the International Classification of Functioning, Disability and Health (ICF; World Health Organization 2001). The ICF Core Sets for ASD include categories emphasizing activities of daily living and functioning in various environmental contexts for persons with ASD encompassing both strengths and challenges associated with ASD, and their impact on participation in everyday activities. The ICF Core Sets for ASD can therefore serve as a foundation for development of more standardized and more personalized assessment tools that could help improve service provision (Bölte et al. 2018). Other well-known measures, although not ASD-specific, could also be appropriate, e.g. the ABILITIES Index (Simeonsson and Bailey 1991).

One of the subscales introduced by Goodman (1999) into the extended version of the SDQ was the one-item burden scale. The concept of burden was defined by Platt (1985) as “the presence of problems, difficulties or adverse events which affect the life (lives) of the psychiatric patients’ significant other(s), e.g. members of the household and/or the family” (p. 385). As part of our study, we looked at possible relationships between grandparents’ needs and grandparents’ perceived burden of grandchild’s difficulties on them or their families. Regression analyses revealed a positive association between felt burden imposed by their grandchild’s ASD and their perception of needs. This finding could be partly explained by impact that severity of grandchild’s ASD might have on family’s everyday functioning as previous empirical research on parental needs has suggested that perceptions of family needs may vary as a function of type and severity of disability (Epley et al. 2011). The finding is also congruent with earlier research demonstrating that child’s symptomatology (diagnosis combined with impairment) significantly predicted perceived burden in parents (Angold et al. 1998). However, this explanation would contradict our results from descriptive statistical analyses, demonstrating that overwhelming majority of the grandparents (91.1%) did not actually perceive their grandchild’s difficulties as a burden on them or family as a whole, whereas only nine respondents (8.9%) did so. These findings point to a complex picture of relationships between experiences of burden in family members and their perceptions of difficulties caused by child’s ASD, and therefore, requires further investigation. As Angold et al. (1998) noted, “It would also be interesting to know what criteria parents use in deciding to attribute a difficulty of their own to a child’s behavior, and why some parents report no burden despite having a severely disturbed child” (p. 79). In this connection, most recent findings drawn from the area of stress research with families of children with ASD could be informative. For instance, a study by Sim et al. (2018) showed no direct associations between severe family stress and child’s characteristics, such as ASD diagnosis. The authors recommended that in order to understand patterns of stress management in families of children with autism, one should study the influence of resilience factors, such as informal social support and respite care, in the context of the child’s whole family system nested in the broader social environment. Indeed, as evidence suggests, presence of resources such as self-differentiation and social support can be associated with reduced stress in grandparents irrespective of their grandchild’s disability status (Findler 2014). Among other protective factors that can contribute to resilience and personal growth in grandparents of children with disabilities are intergenerational connections and the grandparenting role with the accompanying feelings of unconditional love, joy, and gratitude (Findler 2014; Hillman et al. 2017). Grandparents’ positive feelings and satisfaction with their role are pivotal in the family system where a child with ASD is present: grandparents can be invaluable resource to parents and siblings by providing practical and emotional support (Pit-ten Cate et al. 2007; Prendeville and Kinsella 2018).

Previous research with grandparents of children with autism has reported on grandmothers’ greater involvement in grandchild care compared to grandfathers (Glasberg and Harris 1997; Hillman et al. 2016). Several authors argued that grandchild care is a gendered experience entailing roles, expectations and activities different for grandmothers and grandfathers (Di Gessa et al. 2016b). As Hoffman and Mitchell (1998) noted, women and caregiving task are still seen as synonymous notions as historically many women have devoted their lives to this role. Although the results of the present study showed similar amount of needs for both grandmothers and grandfathers, we inquired about the influence of the gender variable on the observed relationship between grandparents’ perceived burden discussed earlier; we predicted that women would feel more burden compared to men. However, the results of the Kruskal–Wallis test showed no differences in perceptions of burden between grandmothers and grandfathers. This finding might suggest that grandparents’ roles in families with a child with autism may have changed over time and can vary in different cultural contexts. For instance, in Ireland, grandfathers’ role has been described as having the calming influence on family, especially in those families where a grandchild with ASD had significant behavioral problems (Prendeville and Kinsella 2018). In Sweden, a country characterized by a gender equality (Kolk 2014), gender differences in relation to childcare are not vivid: nowadays both mothers and fathers are equally engaged in their children’s lives (Kridahl 2017). As Kridahl (2017) suggested, perceptions and expectations of grandmothers’ and grandfathers’ roles in the Swedish family may have disappeared over time and, therefore, may be similar to grandchild’s parents, especially given the fact that Swedish grandparents are not primary caregivers for their grandchildren. However, due to the lack of research on grandparenting a child with ASD in Sweden, unfortunately, it is not possible to draw any firm conclusions at the moment; more research is needed to obtain first-hand perspectives of both grandparents and their adult children on perceptions of roles of grandmothers and grandmothers when caring after a young child with ASD. Longitudinal research designs could help elucidate possible changes in these roles over time in Sweden as well as in other cultural contexts.

### Implications for Practice

The results of our study have important implications for professionals involved into provision of early intervention programs for children with ASD and their family members. The findings can inform practitioners about grandparents’ most and least prioritized needs in various topic areas, which may help practitioners improve already existing formal support programs offered to grandparents, parents, and preschool teachers. For instance, meeting grandparents’ need of information about how to help develop grandchild’s skills can potentially assist grandparents to become more active in supporting of their grandchild. This is especially salient if the child’s parents have full-time jobs. Also, as the results of the present study suggest, grandparents expressed a need for meeting their grandchild’s teachers. It would be desirable if practitioners at disability services could inform preschool teachers about those needs and encourage them to view grandparents as potential partners when providing educational interventions. As Findler’s (2007) study showed, teachers and special educators rarely met grandparents of children with disabilities in their practice; moreover, they expressed no interest in receiving special training on how to meet the needs of grandparents. Existing pre-service general and special educational programs could include this component in their curricula in order to raise educators’ awareness of the beneficial role of grandparents in supporting the child with ASD, his/her siblings and parents, as well as other family members in a broader family system network.

Provision of publicly funded psychoeducational workshops by disability services in Stockholm specifically designed for grandparents as part of family-centered early intervention programs for children with ASD can serve as an example of a support model tailored to the needs of extended family members. This model adds to the list of available workshop models for grandparents of children with disabilities described in the literature (e.g. Vadasy et al. 1986). However, future research needs to evaluate the effectiveness of these models (Kahana et al. 2015; Seligman and Darling 1997).

### Limitations of the Study

The findings of this study should be seen in the light of its limitations. Firstly, the sample in our study was not representative in terms of socio-demographic characteristics: the majority of the grandparents held university academic degree and spoke Swedish as their mother language. Obtaining a more representative sample of grandparents from various educational, socio-economic and cultural backgrounds is important for research and practice (Hillman et al. 2017; Zakirova Engstrand et al. 2018). Involving ethnically and socio-economically diverse participants ensures external validity of research findings (Kistner and Robbins 1986; Pierce et al. 2014; West et al. 2016) and help develop culturally competent services for families of children with ASD (Stahmer et al. 2011; Zakirova Engstrand et al. 2018). Moreover, our findings may not be generalizable to other cultural settings due to differences in provision of formal supports across different countries. Other important limitations are related to methodological aspects, particularly in relation to the extended version of the SDQ, (Goodman 1999). Firstly, the SDQ was developed primarily for screening for possible mental/psychiatric disorders to get services or for prevention in a community sample (Goodman 1997, 1999). In our study, the children had already obtained the formal diagnosis of ASD and were already receiving support services, thus constituting a clinical group. Secondly, as we mentioned earlier, the parent version of the SDQ Impact supplement scale was used in the study, which resulted in a rather large number of missing data for the items eliciting answers on child’s impairment in social life. In fact, during the data collection phase, some of the grandparents told to the first author that they could not answer the survey questions as they did not actually live with their grandchild in the same house. Thirdly, the burden item included into the SDQ Impact supplement scale did not clearly delineate between perceptions of burden for each individual grandparent, and for family as a whole. For future research, it is recommended using more objective instruments to investigate the longer-term effects of grandchild disability on grandparental functioning (Hastings 1997).

Despite these limitations, the study does provide valuable information on how to improve already existing support programs for children with ASD and their family members, and facilitate better planning of future support services based on identified needs. Furthermore, as research on needs of non-custodial grandparents of children with ASD is scant, the present study contributes to literature by providing additional evidence to understand the multi-dimensional nature of issues related to grandchild care in the context of childhood disability.

## Electronic supplementary material

Below is the link to the electronic supplementary material.

Supplementary material 1 (DOCX 18 KB)
