# Supplementary material for: Needs of Grandparents of Preschool-Aged Children with ASD in Sweden
Source: J Autism Dev Disord. 2019 Mar 1;50(6):1941–57. doi: 10.1007/s10803-019-03946-w (PMC7261267; doi:10.1007/s10803-019-03946-w)
Supplement: Supplementary file 1 — Supplementary material 1 (DOCX 18 KB) [file 10803_2019_3946_MOESM1_ESM.docx]

*Table A*. Mean scores and standard deviations for the SDQ Impact supplement scale items. The last column presents valid numbers of responses for each item.

| ***Item*** | | ***Mean*** | ***SD*** | ***n*** |
| --- | --- | --- | --- | --- |
| **Perceived Difficulties** | | 2.61 | 0.63 | 102 |
| **Chronicity** | | 3.91 | 0.31 | 102 |
| **Impact Score (0-10)** | | 2.79 | 2.66 | 79 |
| 1. | Distress | 0.34 | 0.53 | 79 |
| 2. | Home life | 0.53 | 0.64 | 79 |
| 3. | Friendships | 0.73 | 0.75 | 79 |
| 4. | Learning environments | 0.63 | 0.70 | 79 |
| 5. | Leisure activities | 0.56 | 0.73 | 79 |
| **Perceived Burden on Grandparent or Family** | | 0.58 | 0.65 | 101 |
|  | |  |  |  |

*Table B.* Mean scores and standard deviations for each item of the Grandparent Needs Survey (scale 0-2). The last column presents valid numbers of responses for each item (sample *N*=120).

| ***Item*** | | ***Mean*** | ***SD*** | ***n*** |
| --- | --- | --- | --- | --- |
| **Need for Information** | |  |  |  |
| 1. | More information about my grandchild’s development | 1.83 | 0.44 | 117 |
| 2. | More information about how to play or talk to my grandchild | 1.84 | 0.43 | 119 |
| 3. | More information about how to help my grandchild develop skills | 1.92 | 0.33 | 120 |
| 4. | More information about how to handle my grandchild’s behavior | 1.88 | 0.39 | 119 |
| 5. | More information about my grandchild’s ASD | 1.87 | 0.40 | 119 |
| 6. | More information on services that are presently available for my grandchild | 1.78 | 0.53 | 117 |
| 7. | More information on services that my grandchild might receive in the future | 1.83 | 0.44 | 117 |
| 8. | More information about existing laws regulating service and support provision and my grandchild’s rights to obtain them | 1.68 | 0.61 | 118 |
| **Need for Family and Social Support** | |  |  |  |
| 9. | Talking to my grandchild’s parents about concerns related to my grandchild’s ASD | 0.88 | 0.82 | 119 |
| 10. | Having more friends to talk to | 0.61 | 0.73 | 116 |
| 11. | Finding more time for myself | 0.32 | 0.60 | 115 |
| 12. | Helping my spouse accept our grandchild’s ASD | 0.20 | 0.51 | 115 |
| 13. | Helping my grandchild’s parents accept their child’s ASD | 0.38 | 0.62 | 119 |
| 14. | Helping our family discuss problems and reach solutions | 0.85 | 0.80 | 119 |
| 15. | Helping our family support each other during difficult time | 0.84 | 0.80 | 117 |
| 16. | Deciding who will do household chores, child care, and other family tasks | 0.28 | 0.55 | 116 |
| 17. | Choosing and participating in joint family recreational activities | 0.44 | 0.67 | 114 |
| 18. | Helping our family cope with separation or divorce | 0.25 | 0.62 | 112 |
| **Financial Support** | |  |  |  |
| 19. | Paying for expenses such as food, housing, medical care, clothing or transportation | 0.23 | 0.57 | 115 |
| 20. | Getting any special equipment my grandchild needs | 0.48 | 0.71 | 115 |
| 21. | Paying for therapy, or other services my grandchild needs | 0.38 | 0.63 | 112 |
| 22. | Counselling or help in getting a job for myself or spouse | 0.18 | 0.51 | 110 |
| 23. | Paying for child care (i.e. babysitting) or respite care | 0.23 | 0.55 | 111 |
| 24. | Paying for toys or recreation that my grandchild needs | 0.31 | 0.60 | 110 |
| **Explaining to Others** | |  |  |  |
| 25. | Explaining my grandchild’s disability to my relatives | 0.63 | 0.77 | 115 |
| 26. | Explaining my grandchild’s disability to his or her siblings | 0.43 | 0.71 | 115 |
| 27. | Knowing how to respond when friends, neighbors or strangers ask about my grandchild | 0.76 | 0.80 | 115 |
| 28. | Explaining my grandchild’s disability to other children | 0.77 | 0.83 | 115 |
| 29. | Finding reading material about families who have a grandchild like mine | 1.04 | 0.81 | 115 |
| **Child Care** | |  |  |  |
| 30. | Learning special skills needed to care for my grandchild | 1.40 | 0.76 | 114 |
| 31. | Learning how to provide adaptive play or recreation experiences for my grandchild | 1.60 | 0.59 | 113 |
| 32. | Learning how to include my grandchild with ASD in grandparent child care time along with my other grandchildren | 1.40 | 0.78 | 113 |
| 33. | Locating appropriate child care (e.g. preschool) for my grandchild with ASD | 0.41 | 0.72 | 113 |
| **Professional Support** | |  |  |  |
| 34. | Meeting with a leader of my religious faith | 0.02 | 0.18 | 116 |
| 35. | Meeting with a counselor (e.g. psychologist, social worker, psychiatrist) | 0.46 | 0.69 | 116 |
| 36. | Learning how to communicate with teachers and other professionals regarding my grandchild with ASD | 0.65 | 0.74 | 116 |
| 37. | Attending a support group to talk with other families who have similar needs | 0.57 | 0.71 | 116 |
| 38. | Accessing family counselling for parents and grandparents | 0.65 | 0.78 | 110 |
| **Community Service** | |  |  |  |
| 39. | Locating a dentist who can provide dental care to my grandchild with ASD | 0.28 | 0.58 | 116 |
| 40. | Locating a family doctor or a specialist doctor who can understand my grandchild’s needs | 0.41 | 0.64 | 116 |
